# A Decision for Predicting Successful Extubation of Patients in Intensive Care Unit

**DOI:** 10.1155/2018/6820975

**Published:** 2018-01-04

**Authors:** Chang-Shu Tu, Chih-Hao Chang, Shu-Chin Chang, Chung-Shu Lee, Ching-Ter Chang

**Affiliations:** ^1^Department of Information Management, Chang Gung University, Taoyuan City, Taiwan; ^2^Department of Thoracic Medicine, Chang Gung Memorial Hospital, Linkou, Taoyuan, Taiwan; ^3^Division of Pulmonary and Critical Care, Department of Internal Medicine, Saint Paul's Hospital, Taoyuan, Taiwan; ^4^Department of Accounting, Chung Yuan Christian University, Taoyuan, Taiwan; ^5^Department of Industrial Engineering and Management, Ming Chi University of Technology, New Taipei, Taiwan

## Abstract

Approximately 40% of patients admitted to the medical intensive care unit (ICU) require mechanical ventilation. An accurate prediction of successful extubation in patients is a key clinical problem in ICU due to the fact that the successful extubation is highly associated with prolonged ICU stay. The prolonged ICU stay is also associated with increasing cost and mortality rate in healthcare system. This study is retrospective in the aspect of ICU. Hence, a total of 41 patients were selected from the largest academic medical center in Taiwan. Our experimental results show that predicting successful rate of 87.8% is obtained from the proposed predicting function. Based on several types of statistics analysis, including logistic regression analysis, discriminant analysis, and bootstrap method, three major successful extubation predictors, namely, rapid shallow breathing index, respiratory rate, and minute ventilation, are revealed. The prediction of successful extubation function is proposed for patients, ICU, physicians, and hospital for reference.

## 1. Introduction

In recent years, human activities, such as burning of fossil fuels and coal, have led to dust-storm, frog, and haze [1]. Several epidemiological studies have shown the effects of chronic exposure to air pollution (e.g., PM_2.5_, nitrogen dioxide, and NO_2_) on lung function [2]. Air pollution is closely related to both the development and exacerbation of pulmonary disease. In the worst case, approximately 40% of all pulmonary disease patients in medical intensive care unit (ICU) require mechanical ventilation [3, 4]. Many of them are extubated in 2 to 4 days after the start of ventilation, whereas up to 25% require mechanical ventilation for more than 7 days [5]. In spite of weaning protocols, automated systems, daily spontaneous breathing trials, and pressure-support ventilation, it is estimated that 20–30% of patients cannot be extubated upon the first weaning attempt [6]. 29% met the criteria for extubation failure [7], and 40% extubation failure was found in acute ischemic stroke patients [8].

The rapid shallow breathing index (respiratory frequency/tidal volume, *f*/*V*_*T*_) and spontaneous breathing trials (SBT) have been recognized as useful markers in predicting successful weaning from mechanical ventilation. However, they are imperfect, and clinicians always incorporate other factors for final extubation decision. The traditional extubation decision is solely based on expert clinical judgment. For instance, continuous positive airway pressure could be tolerated at 5 to 7 cm H_2_O without fatigue for 12 hours, arterial PO_2_ > 80 mmHg on room air, and bulbar paresis improved. Some studies have predicted the timing of extubation [9]. The factor predicting extubation success in patients in neurocritical care units is addressed [10], and early predictors of extubation success in acute ischemic stroke are studied [8]. Farghaly and Hasan [11] proposed diaphragm ultrasound as a new method to predict extubation outcome in mechanically ventilated patients. A prospective observational cohort study is performed to predict extubation failure after successful completion of a SBT [12]. Zettervall et al. [13] aimed to evaluate the effect of extubation time on patient's outcomes after endovascular aneurysm repair and open repair. Miu et al. [14] proposed two prediction models for extubation failure in subjects who have passed an SBT: one for failure at any time and another for failure in the first 24 hours after extubation. Savi et al. [15] evaluated the potential of weaning predictors during extubation. All of above-mentioned methods are not always precise. Therefore, a precise prediction of successful extubation for patients is an important issue and worthy of study. A delay in extubation can increase the risk of ventilator-related complications such as pneumonia, tracheobronchitis, or barotrauma. A premature extubation may lead to the necessity of reintubation with an associated increase in the risk of ventilator-associated pneumonia and airway trauma. The delay or premature extubation may lead to prolonged ICU stay [16]. The prolonged ICU stay is also associated with increased cost and decreased mortality rate in healthcare systems. Therefore, accurate prediction of successful extubation is a key clinical problem. However, no prior work has been done to provide a good prediction function of successful extubation for decision making. In order to provide more precise prediction of successful extubation in ICU, the retrospective study is conducted. Hence, the purpose of this study is to find the key predictors of the successful extubation in critically ill patients. In addition, this study aims to develop a prediction function of successful extubation for effective decision making of extubation

## 2. Material and Methods

### 2.1. Data Collection

**Figure 1:**
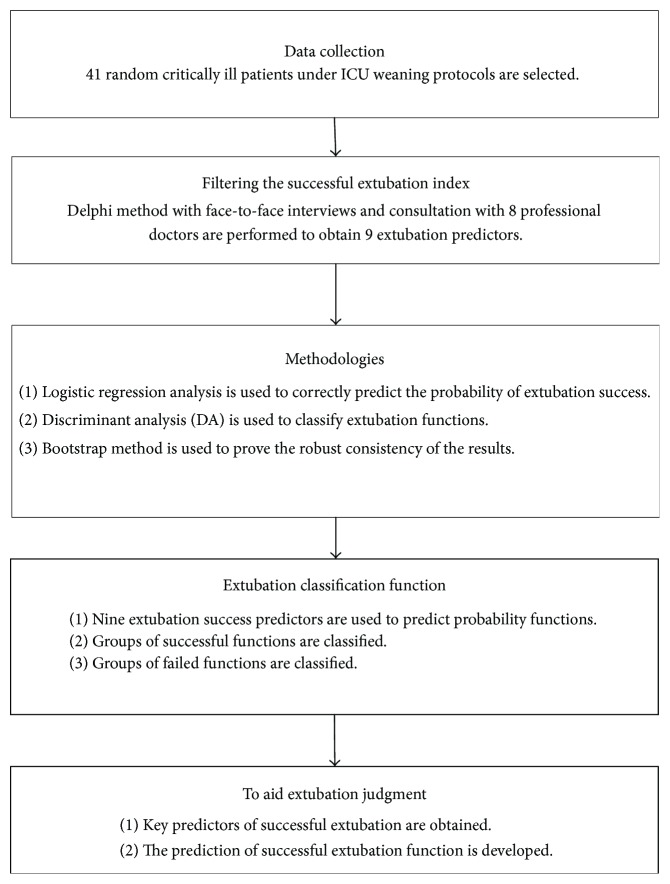
Experimental procedure.

This study was conducted at the Chang Gung Memorial Hospital (CGMH) in Taiwan that was approved by the Institutional Review Board (number 103-6085B) of the hospital. CGMH is one of the world's leading medical centers and currently Taiwan's largest hospital with 3700 beds. In order to meet the requirements of medical services, CGMH has set up many hospitals in Taiwan and China. For this study, 10 weaning indices are reviewed and analyzed, as shown in Table 1. 41 critically ill patients under ICU weaning protocols are randomly selected. Although the sample size is relatively small, the minimum acceptance level is defined as 30 samples [10, 11]. The mean age was 74 ± 2 years. 27 patients were men (65.8%) and 14 were women (34.1%). Other demographic details are listed in Tables 2 and 3. First, we discussed assessment success factors and weights of extubation with professional doctors. Then, we obtained necessary critical data for all patients from ICU staff screening to facilitate the experimental study. In order to filter out successful extubation predictors, following weaning protocols, we used the Delphi method with face-to-face interviews and consultation with 8 professional doctors in the department of chest diseases at CGMH, who helped to obtain the most important 9 successful extubation predictors, such as (1) gender; (2) Glasgow Coma Scale (GCS): E (eye), V (verbal), and M (motor) score; (3) respiratory rate (RR) (*f*); (4) minute ventilation (MV); (5) maximal inspiratory pressure (PiMax or MIP); (6) rapid shallow breathing index (RSBI); (7) arterial blood gas (ABS) and PH; (8) arterial carbon dioxide tension (PaCO_2_); and (9) partial pressure of oxygen (PaO_2_). In order to solve this problem, an experimental procedure is conducted, as shown in Figure 1.

### 2.2. Statistical Analysis

Among the 41 patients included in this study, 23 (56%) were successfully extubated. All statistical analyses are considered significant when *p* < 0.05 in two-tailed *t*-tests. Statistical calculations are performed using the IBM Statistical Package for the Social Sciences (SPSS) software. The extubation failure is defined as reintubation within 48 hours of extubation.  Table 4 shows the successful extubation as well as extubation failure groups. Three predictors, GCS, MV, and RSBI, reach the significance level of 0.05. To find the relative importance weights among the 9 successful extubation predictors, multivariate logistic regression analysis is used to obtain a correlation matrix, as shown in Table 5. Then, unstandardized beta coefficient values (*y*), −0.049,0.569, −0.046,0.151, −0.092,0.529, −0.862,0.302, and −0.307, and standardized beta coefficients values (*y*), −0.047, 0.560, −0.592,0.930, −0.055,0.664, −0.094,0.146, and −0.140, are used in the multiregression correlation matrix to obtain the rescaled relative weights, as shown in Table 6. Based on the method proposed by Braun and Oswald [33], the standardized beta coefficients and rescaled relative weighting values of RSBI (32.5%), RR (22%), and MV (18%) were found as the top three predictors. Others predictors are gender (10%), PaO_2_ (8%), PH (6%), GCS (2%), PiMax (1%), and PaCO_2_ (0%).

### 2.3. Logistic Regression Analysis

Logistic regression analysis is used to obtain the rate of successful extubation, as shown in Table 7. The overall model is significant, *χ*^2^ = 24.516 (*p* value = 0.004 < 0.05), while the Hosmer and Lemeshow test = 16.17 (*p* value = 0.04 < 0.05) reached a significant level. Cox-Snell *R*^2^ = 0.450 and Nagelkerke *R*^2^ = 0.603. The results show that moderate association exists. The Wald values of GCS, MV, and RSBI are 6.261, 4.094, and 3.009, respectively. The* p* values of GCS, MV, and RSBI equal 0.012, 0.043, and 0.083 (close to 0.05), respectively. The odds ratios of three key predictors, namely, GCS, MV, and RSBI, are 0.029, 8.011, and 7487.943, respectively. Thus, the prediction of successful extubation function (PSEF) can be obtained as

(1) lnpi1−pi=0.058∗Gender  –  3.551∗GCS  –  0.758∗RR+2.081∗MV+0.471∗PiMax+8.921∗RSBI  –  8.891∗PH+3.818∗PaO2  –  2.855∗PaCO2+23.404,

where ⁡*p*_*i*_ is the probability of successful extubation.

The PSEF is solved using IBM SPSS software syntax to obtain the successful rate of the observation as 87.8%, as shown in Table 9. As a result, a high accuracy rate of the PSEF is obtained. Only 5 observations are misjudged. The reasons for classification of logistic regression analysis are shown in Table 8. The values of sensitivity, specificity, positive predictive, negative predictive, false positive rate, and false negative rate can be obtained as 0.957, 0.778, 0.846, 0.933, 0.153, and 0.066, respectively. This indicates that the PSEF has a high rate of successful classification.

### 2.4. Comparing the Classification Rates of the Two Methods

The summary of classifications of 9 extubation predictors by discriminant analysis and logistic regression analysis is used to validate the classification results, as shown in Table 10. The logistic regression analysis offers a successful classification rate of 80.5% and discriminant analysis gives a successful classification rate of 87.8% for prediction of extubation.

### 2.5. Discriminant Classification Function

The factor of the successful extubation predictors for the classification of Fisher's linear discriminant functions (see Table 11) affects the PSEF. The fail function of classifying groups is given as follows:

(2) D1=fx=−152.053∗Gender+100.671∗GCS+3.258∗RR+16.664∗MV+172.336∗PiMax+45.964∗RSBI+3305.766∗PH  –  3.363∗PaO2+156.543∗PaCO2  –  13085.09,

and the success function of the classifying groups is given as

(3) D2=fx=−152.399∗Gender+104.655∗GCS+2.940∗RR+17.718∗MV+171.695∗PiMax+49.667∗RSBI+3299.738∗PH  –  1.253∗PaO2+154.393∗PaCO2  –  13057.793.

**Figure 2:**
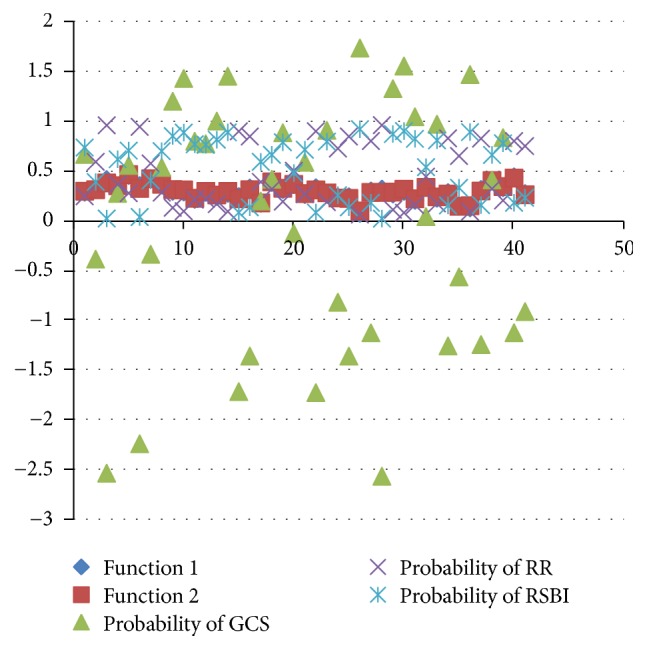
Scatter plot for the top three predictors of the classification functions.

**Figure 3:**
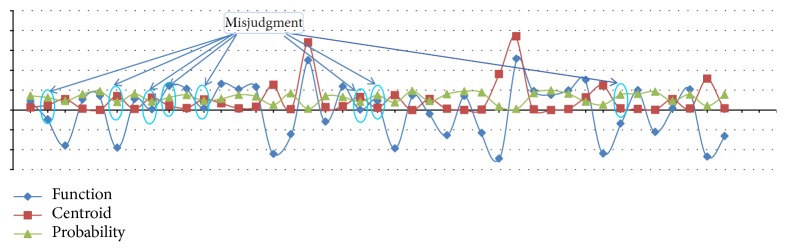
Misjudgment analysis in discriminant classification function.

**Figure 4:**
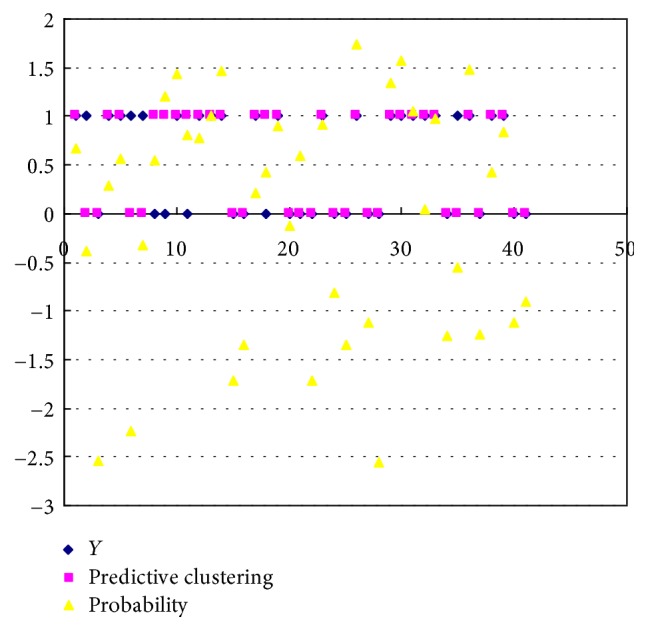
Predictive clustering scatter plot by discriminant analysis.

IBM SPSS software with Fisher's linear discriminant function is used to obtain the probability values.  Figure 2 shows the scatter plot of the probability of classification for top three predictors in different functions, when the default cut-off point is 0.5 and the prediction probability is greater than 0.5. Top three predictors reveal very little difference in the distance (0 and 1). It is observed that the classification function can be accurately predicted.  Figure 3 shows the 8 misjudged observations and 33 correctly predicted observations in discriminant classification function. In order to verify the correctness of the classification of Fisher's linear discriminant functions, we use Press' *Q* formula to test the predictability of the clustering results and get a Press' *Q* value = 15.24 > 3.84. Indeed, it is a good classification. As seen in Figure 4, there are four misclassified samples (which actually should be attributed to fail extubation group). There are four other misclassified samples, which actually should be attributed to the successful extubation group [34].

Therefore, the bootstrap method is used to reduce the gap between the sample data and the general population by estimating path coefficients repeatedly [35]. Bootstrapping is also undertaken to confirm the robustness of the findings. To do this, our study uses the IBM SPSS of bootstrap method [36] to generate 1000 random numbers. Bootstrap samples were built by resampling with replacement of the original sample. Finally, the repeating presentation pattern of the sampling results shows robust consistency. The bootstrapping method is commonly used to calculate confidence intervals around the success indexer estimates. The summary results for bootstrapping are provided in Table 12. The critically ill patient's data of 41 repeated samples (using the bootstrap method) are as follows: GCS = 0.009, MV = 0.023, and RSBI = 0.053. Only three successful extubation predictors RR, MV, and RSBI are significant. 15 critically ill patient's data types are used again by the bootstrap method to obtain the following: RR = 0.014; MV = 0.014; RSBI = 0.014; PH = 0.014; PaO_2_ = 0.014; PaCO_2_ = 0.014. 6 successful extubation predictors RR, MV, RSBI, PH, PaO_2_, and PaCO_2_ are significant.

## 3. Discussion

RSBI < 105 has 90% sensitivity, whereas 18% specificity was found. Artificial neural network has been used to predict extubation outcome although its result varies in different studies [37, 38]. Miu et al. [14] proposed a few important risk factors for extubation failure. For instance, oxygenation was an important component of early failure. Lower diastolic blood pressure and repeatedly failed SBT are significant contributors to extubation failure at any time. Two prediction models for extubation failure are found in subjects who have passed an SBT: one for failure at any time and another for failure in the first 24 hours after extubation. Approximately, both models showed 70% accuracy when correct predicting was obtained. Nguyen et al. [10] found that lower negative inspiratory force and higher vital capacity are corrected with successful extubation. SBT is the major diagnostic test to determine whether patients can be successfully extubated [39]. Lioutas et al. [8] indicated that conventional respiratory parameters have no effect on extubation success in acute ischemic stroke patients. The PaCO_2_ appears as a strong predictor of extubation failure [12]. However, all of the above-mentioned methods are not always precise and do not provide a decision function for aiding the decision making of extubation.

Except weaning predictors, some clinical rules such as mental status and endotracheal secretions are used to predict extubation failure [40]. Muscle weakness resulting from critical illness polyneuropathy or myopathy causes failure to wean from the ventilator. Farghaly and Hasan [11] proposed diaphragm ultrasound as a new method to predict extubation outcome in mechanically ventilated patients. Besides, prolonged mechanical ventilation is defined as greater than 21 days of mechanical ventilation for at least 6 hours per day. Failed weaning had a lower hypercapnic ventilatory response than successfully weaned subjects [41].

In this retrospective study, we found a more precise function for prediction of successful extubation in ICU of CGMH. Our experimental results show that predicting successful rate of 87.8% is obtained from the proposed predicting function. Multiple statistical methods are used to obtain the prediction of successful extubation function, *D*_2_. In addition, the bootstrap method is used to confirm the robustness of the findings. Top three predictors, namely, RSBI (32.5%), RR (22%), and MV (18%), are found for successful extubation in ICU of CGMH. The prediction of successful extubation function is also provided for aiding the clinicians to make a more precise extubation decision for patients in ICU to avoid delay or premature extubation against the potential harms of patients. This decision is very important because the failed extubation is associated with worse patient outcome.

## 4. Limitations

Our study has several limitations: (1) overoptimistic estimate of the predictive performance is a problem because a small size of the data set is used in this retrospective study; (2) an external validation can be used to enhance robustness of the prediction of successful extubation function [42]; (3) not all clinical weaning predictors, such as diaphragm movement, endotracheal secretions, or hypercapnic ventilatory response were collected in each patient; (4) the number of patients in this retrospective study was relatively small. However, the study emphasized the methodologies for the weaning predictors. We recommend that further studies are needed to evaluate larger samples of respiratory ICU patients.

## 5. Conclusion 

The results show several strengths of relative weights. First, relative weights add up to *R*^2^ [36]. Additionally, relative weights are easy to explain to researchers [43]. Second, three major predictors of success of extubation are found in both discriminant analysis and logistic analysis. A successful classification rate of 87.8% was obtained to avoid delay or premature extubation against the potential harms and costs of failed extubation. The prediction of successful extubation function was derived, which can easily be used to aid clinical extubation judgment. Our study is a monocentric retrospective pilot trial involving limited number of critically ill patients. Further studies are needed in terms of larger and more heterogeneous patient groups to precisely revise the coefficients of PSEF.

## Figures and Tables

**Table 1:** The literature review of successful extubation predictors.

Extubation predictors	Description	Sources
Tidal volume (*V*_*T*_)	Tidal volume is the *lung volume* representing the normal volume of air displaced between normal inhalation and exhalation when extra effort is not applied.	Epstein, 1995 [17] Newth et al., 2009 [18] Nemer et al., 2009 [19] Adigüzel et al., 2009 [20]

Respiratory rate (*f*) (RR)	The respiratory rate (RR) is also known as the respiration rate, ventilation rate, ventilatory rate, ventilation frequency (*V*_*f*_), respiration frequency (*R*_*f*_), pulmonary ventilation rate, or breathing frequency.	Yang and Tobin, 1991 [21] Sassoon and Mahutte, 1993 [22] Epstein, 1995 [17]

Minute ventilation (MV)	The total lung ventilation per minute is the product of tidal volume and respiration rate. It is measured by expired gas collection for a period of 1 to 3 minutes. The normal rate is 5 to 10 liters per minute.	Epstein, 1995 [17]

Rapid shallow breathing index (RSBI)	The rapid shallow breathing index (RSBI) is a tool that is used in the weaning of *MV* in *intensive care units*. The RSBI is defined as the ratio of *respiratory frequency* to tidal volume (*f*/*V*_*T*_).	Tanios et al., 2006 [23] Chang et al., 2007 [24] Rodriguez and Varon, 2008 [25] Nemer et al., 2009 [19] Newth et al., 2009 [18] Fadaii et al., 2012 [26]

Maximal inspiratory pressure (PiMax or MIP)	Maximum inspiratory pressure (MIP) is a measure of the strength of respiratory muscles, obtained by making the patient inhale as strongly as possible with the mouth against a mouthpiece; the maximum value is near the residual volume.	Nava et al., 1994 [27] de Souza et al., 2013 [28]

Arterial carbon dioxide tension (PaCO_2_)	A measure of the partial pressure of carbon dioxide in the arterial blood.	Mokhlesi et al., 2007 [9] Nemer et al., 2009 [19] de Souza et al., 2013 [28]

Partial pressure of oxygen (PaO_2_)	When measuring *arterial blood gases*, we sometimes use the term partial pressure of oxygen or PaO_2_. Partial pressure refers to the pressure exerted by a specific gas in a mixture of other gases. PaO_2_, put simply, is a measurement of oxygen in arterial blood. The normal range for PaO_2_ is 75–100 mm Hg. If a patient's PaO_2_ is less than this, it means that he/she is not getting enough oxygen.	El Khoury et al., 2010 [29]

Static compliance of the respiratory system (Cst, rs)	The static compliance of the respiratory system (Cst, rs) is measured using volume control ventilation.	Nemer et al., 2009 [19]

Time inspiratory effort (TIE)	The timed inspiratory effort (TIE) index was developed based on the premise that patients with poor neuromuscular efficiency need more time to develop a maximal effort during the occlusion maneuver.	de Souza et al., 2013 [28]

Arterial blood gas (ABS)	Arterial blood gas (ABG) is a *blood test* that is performed using *blood* from an *artery*.	Murphy et al., 2006 [30] Zavorsky et al., 2007 [31] Khan et al., 2010 [32]

**Table 2:** Demographic details of critically ill patients.

Total number of patients	41
Age (mean ± SD), years	74 ± 2.495
Gender (M/F)	27/14
Successful, failed extubation	23/18
Glasgow Coma Scale (GCS) E (mean ± SD)	3.71 ± 0.106
Glasgow Coma Scale (GCS) V (mean ± SD)	4.44 ± 0.148
Glasgow Coma Scale (GCS) M (mean ± SD)	5.54 ± .149
RR (*f*) (mean ± SD)	23 ± 1.021
Tidal volume (mean ± SD)	0.312 ± 0.0283
Minute ventilation (mean ± SD)	6.568 ± 0.4845
PiMax (MIP, NIF) (mean ± SD)	−37.88 ± 2.202
RSBI (*f*/TV) (mean ± SD)	99.98 ± 10.355
Arterial blood gas pH (mean ± SD)	7.44 ± 0.0085
PaO_2_ (mean ± SD)	112.076 ± 4.279
PaCO_2_ (mean ± SD)	41.944 ± 1.5901

**Table 3:** Underlying diseases patient characteristics.

Chronic obstructive pulmonary disease (COPD)	6 (15%)
End stage renal disease (ESRD)	2 (5%)
Old stroke	8 (20%)
Cervical cancer	1 (2%)
Cirrhosis	2 (5%)
Hepatitis B virus (HBV)	1 (2%)
Heart failure	3 (7%)
Esophageal cancer	2 (5%)
Prostate cancer	2 (5%)
Hypertension	1 (2%)
Pneumoconiosis	1 (2%)
Hepatocellular carcinoma (HCC)	1 (2%)
Congestive heart failure (CHF)	1 (2%)
Cerebral palsy	1 (2%)
Systemic lupus erythematosus (SLE)	2 (5%)
Colon cancer	1 (2%)
Deep vein thrombosis (DVT)	1 (2%)
Old tuberculosis (TB)	1 (2%)
Pulmonary tuberculosis (TB)	1 (2%)
Renal cell carcinoma (RCC)	1 (2%)
Nil	2 (5%)
Total	41 (100%)

**Table 4:** Tests of successful and failed extubation groups.

Extubation index	Successful extubation (*n *= 23)	Failed extubation (*n* = 18)	*p* value (two-tailed)
Gender	0.78 ± 0.088	0.50 ± 0.121	0.06
GCS	0.83 ± 0.081	0.33 ± 0.114	0.001^*∗∗*^
RR	22.70 ± 1.335	23.39 ± 1.591	0.741
MV	7.497 ± 0.723	5.381 ± 0.495	0.028^*∗*^
PiMax	−40.61 ± 3.378	−34.39 ± 2.417	0.126
RSBI	85.48 ± 11.559	118.50 ± 17.825	0.05^*∗*^
PH	7.44 ± 0.010	7.46 ± 0.014	0.296
PaO_2_	112.81 ± 6.162	111.13 ± 5.939	0.968
PaCO_2_	39.89 ± 1.706	44.567 ± 2.83	0.170

^*∗*^ *p* < 0.05; ^*∗∗*^*p* < 0.01.

**Table 5:** Multivariate regression correlation matrix.

	PaCO_2_	PH	RSBI	PaO_2_	GCS	Gender	PiMax	MV	RR
PaCO_2_	1								
PH	0.126	1							
RSBI	−0.109	−0.078	1						
PaO_2_	−0.050	−0.148	0.175	1					
GCS	0.209^*∗*^	0.250	0.205^*∗*^	−0.078	1				
Gender	−0.229^*∗*^	−0.339	−0.015^*∗*^	0.140	−0.566^*∗*^	1			
PiMax	−0.082	0.167	−0.015	0.035	−0.273	0.326	1		
MV	0.001^*∗*^	0.006	0.879^*∗*^	0.212	0.341^*∗*^	−0.293^*∗*^	−0.270	1.	
RR	0.118	0.143	−0.939	−0.174	−0.101	−0.004	0.098	−0.870	1

^*∗*^ *p* < 0.05.

**Table 6:** Rescaled relative weights of successful extubation indexes.

Rescaled relative weights (%)	Gender	GCS	RR	MV	PiMax	RBSI	PH	PaO_2_	PaCO_2_
Unstandardized beta coefficients	21.100	2.651	9.973	8.578	25.889	10.727	19.960	0.728	0.391
Standardized beta coefficients	10.147	2.319	22.078	17.828	0.664	32.545	6.129	7.939	0.346

**Table 7:** The results of logistic regression analysis.

Variable name	*B*	SE	Wald value	Odds ratio	Effect value
Gender	0.058	1.429	0.002	1.059	Cox-Snell *R*^2^ = 0.450 Nagelkerke *R*^2^ = 0.603
GCS	−3.551	1.419	**6.261**	**0.029**	
RR	−0.758	0.447	2.881	0.468	
MV	2.081	1.028	**4.094**	**8.011**	
PiMax	0.471	2.233	0.045	1.602	
RSBI	8.921	5.143	**3.009**	**7487.943**	
PH	−8.891	12.157	0.535	0.000	
PaO_2_	3.818	2.661	2.059	45.520	
PaCO_2_	−2.855	2.388	1.430	0.058	
Constant	23.404	100.915	0.054	14596256529	

Overall pattern match verification	*χ*^2^ = 24.516				
	Hosmer and Lemeshow test = 16.17 significance				

**Table 8:** The reasons for classification of the logistic regression analysis.

Patient number	*y* = extubation	Forecast groups	Predicting probability	classification score	Misjudgment	Analysis of reasons
(1)	1	1	0.756	−2.36		
(2)	1	1	0.545	3.8		
(3)	0	0	0.119	1.5		
(4)	1	1	0.669	−2.78		
(5)	1	1	0.716	−2.67		
(6)	1	0	0.064	0.82	Misjudgment	Positive values should be classified in successful groups
(7)	1	1	0.806	−2.17		
(8)	0	0	0.350	−4.1		
(9)	0	1	0.947	−0.59	Misjudgment	Negative values should be judged as failed groups
(10)	1	1	0.884	−1.45		
(11)	0	0	0.224	−4.85		
(12)	1	1	0.949	−0.56		
(13)	1	1	0.906	−1.22		
(14)	1	1	0.941	−0.71		
(15)	0	0	0.009	−1.16		
(16)	0	0	0.202	2.25		
(17)	1	1	1.000	15.31		
(18)	0	0	0.306	−4.3		
(19)	1	1	0.937	−0.79		
(20)	0	1	0.514	−3.43	Misjudgment	Negative values should be judged as failed groups
(21)	0	1	0.705	−2.61	Misjudgment	Negative values should be judged as failed groups
(22)	0	0	0.048	0.64		
(23)	1	1	0.829	−1.9		
(24)	0	1	0.746	4.58	Misjudgment	Positive values should be classified into successful groups
(25)	0	0	0.113	1.44		
(26)	1	1	0.680	−2.73		
(27)	0	0	0.210	2.18		
(28)	0	0	0.022	−0.28		
(29)	1	1	0.998	2.9		
(30)	1	1	0.896	−1.33		
(31)	1	1	0.881	−1.6		
(32)	1	1	0.917	−1.2		
(33)	1	1	0.979	0.34		
(34)	0	0	0.016	−0.49		
(35)	1	1	0.599	4.02		
(36)	1	1	0.810	−2.04		
(37)	0	0	0.169	1.91		
(38)	1	1	0.523	−3.39		
(39)	1	1	0.877	−1.52		
(40)	0	0	0.003	−2.39		
(41)	0	0	0.135	1.77		

**Table 9:** Classification table for logistic regression analysis.

Observed	Predicted		
	Extubation		Percentage
	Successful	Failed	Correct
Success	22	4	95.7
Fail	1	14	77.8
Overall percentage			87.8

**Table 10:** Summary of classifications of nine successful extubation indexes by two methods.

		Discriminant classification			Logistic regression classification		
	Group	S	F	Classification rate (%)	S	F	Classification rate (%)
Case Extubation	S	87.0	13.0	80.5	95.65	4.35	87.8
	F	27.8	72.2		22.22	77.78	

**Table 11:** The classification of Fisher's linear discriminant functions.

Indexes	Successful (failed) extubation	
	Failed	Successful
Gender	−152.053	−152.399
GCS	100.671	104.655
RR	3.258	2.940
MV	16.664	17.718
PiMax	172.336	171.695
RSBI	45.964	49.667
PH	3305.766	3299.738
PaO_2_	−3.363	−1.253
PaCO_2_	156.543	154.393
Constant	−13085.090	−13057.793

**Table 12:** Bootstrap method with sample analysis of 15 and 41 patients.

Successful index	Solving bootstrap for sample analysis of 15 patients			Solving bootstrap for sample analysis of 41 patients		
	Beta estimates	SE	Significant (two-tailed)	Beta estimates	SE	Significant (two-tailed)
Gender	−0.281	5942.222	0.135	0.058	148.105	0.750
GCS	−0.785	3821.522	0.095	−3.551	208.851	0.009
RR	−0.839	956.320	**0.014**	−0.758	122.741	0.061
MV	2.263	1528.980	**0.014**	2.081	272.902	0.023
PiMax	−2.801	2569.122	0.095	0.471	582.832	0.657
RSBI	9.443	8954.303	**0.014**	8.921	1178.506	0.053
PH	−28.325	16465.99	0.014	−8.891	2084.391	0.387
PaO_2_	5.455	1613.969	0.014	3.818	333.872	0.076
PaCO_2_	−4.553	2601.498	0.014	−2.855	1184.107	0.131
Cox & Snell *R*^2^	0.350			0.450		
Nagelkerke *R*^2^	0.486			0.603		

## Additional Points

*Highlight*. (1) Approximately 40% of patients admitted to the medical intensive care units require mechanical ventilation. (2) Extubation decision solely based on clinical judgment of experts is not always precisely. (3) Our experimental results show that a predicting successful rate of 87% is obtained by the proposed predicting function.* Research Question*. (1) Can a good predicting function for extubation decision be obtained? (2) In practice, what are the successful extubation factors? (3) What is the improvement method for finding more precise predicting function for extubation decisions.
